# MARR-GAN: Memristive Attention Recurrent Residual Generative Adversarial Network for Raindrop Removal

**DOI:** 10.3390/mi15020217

**Published:** 2024-01-31

**Authors:** Qiuyue Chai, Yue Liu

**Affiliations:** 1 School of Electrical and Electronic Engineering, Changchun University of Technology, Changchun 130012, China; 2 Changchun Changding Electronic Technology LLC, Changchun 130012, China

**Keywords:** raindrop removal, recurrent residual network, memristor, attention gate, GAN

## Abstract

Since machine learning techniques for raindrop removal have not been capable of completely removing raindrops and have failed to take into account the constraints of edge devices with limited resources, a novel software-hardware co-designed method with a memristor for raindrop removal, named memristive attention recurrent residual generative adversarial network (MARR-GAN), is introduced in this research. A novel raindrop-removal network is specifically designed based on attention gate connections and recurrent residual convolutional blocks. By replacing the basic convolution unit with recurrent residual convolution unit, improved capturing of the changes in raindrop appearance over time is achieved, while preserving the position and shape information in the image. Additionally, an attention gate is utilized instead of the original skip connection to enhance the overall structural understanding and local detail preservation, facilitating a more comprehensive removal of raindrops across various areas of the image. Furthermore, a hardware implementation scheme for MARR-GAN is presented in this paper, where deep learning algorithms are seamlessly integrated with neuro inspired computing chips, utilizing memristor crossbar arrays for accelerated real-time image-data processing. Compelling evidence of the efficacy and superiority of MARR-GAN in raindrop removal and image restoration is provided by the results of the empirical study.

## 1. Introduction

Adverse weather often results in a significant degradation of captured images [1,2,3,4,5]. As a result, it not only affects the quality of images but also hinders advanced vision tasks, such as semantic segmentation [6], object detection [7], etc. Recently, scholars have devoted considerable attention to the restoration of raindrop-laden images, given their importance in real-world applications [8,9,10]. However, removing raindrops from a single image remains a complex challenge due to the intricate physical structure of these droplets.

Currently, raindrop-removal techniques could be broadly classified into two categories: one is a traditional algorithm based on prior knowledge [11,12], while the other one is based on neural networks [13,14,15,16,17,18,19]. The first method requires a manual design or estimation of the raindrop degradation model of raindrops and relies on prior knowledge to model raindrops. This method, however, exhibits limitations in terms of its generalization ability and computational efficiency. For the past few years, the majority of raindrop-removal techniques have adopted the latter method. A generative adversarial network based on recurrent attention has been suggested by Qian et al. [14], which is used to precisely identify raindrop locations and efficiently remove them from images. To maximize the potential significance of raindrop images, Shao et al. [15] has explored the blurred attributes of raindrops and proposed an uncertainty-driven, multi-scale attention network for enhancing raindrop-affected images. The models generated by this approach exhibit commendable quality. However, they incur the tradeoff from increased model size and reduced inference speed. A rapid and efficient network model has been proposed by Guo et al. [13], wherein the key concept is to treat the rain-removal problem as a pixel-wise filtering task. By leveraging highly optimized filtering operations, accelerated inference speed on the GPU was achieved by this model, while significantly enhancing performance. Nevertheless, additional computational resources and memory consumption are still necessitated for deploying and utilizing this model. In addition, various multi-step structures have been created to improve the overall image restoration performance. These include the cascaded attention guidance network (CAG-Net) [16] and the multi-axis MLP (MAXIM) [17]. Compared to conventional methods, the aforementioned techniques have significantly improved the efficiency and quality of image processing. However, most of these systems rely largely on cyclic and progressive processing modes. As a result, system calculation, inference time, and memory usage all increase significantly. Meeting the requirements for computer vision applications that prioritize efficiency becomes challenging at that point. It also poses plenty of difficulties for the application and usage of rainfall algorithms on various platforms. Therefore, it is imperative to provide a reasoning method for embedded artificial intelligence applications that is more effective.

Deep learning has been greatly influenced by the remarkable contributions of deep convolutional neural networks (DCNNs). These well-constructed DCNNs demonstrated their efficient feature learning ability by successfully extracting abstract information from images of rain. There has been a noticeable enhancement in the quality of de-rain images [20,21,22]. However, the substantial computational and memory demands of DCNN arise from its extensive parameter count and calculations, presenting obstacles for hardware implementation. Additionally, the progress in integrated circuit development has faced limitations, making it challenging to overcome the memory constraints imposed by the von Neumann architecture. Therefore, there has been a significant focus on enhancing hardware implementation and expediting the performance of DCNNs. The memristor, renowned for its nanoscale dimensions, rapid operation, non-volatile data storage capability, low power consumption, programmability, and compatibility with complementary metal–oxide–semiconductor (CMOS) technology [23,24], offers innovative hardware solutions for large-scale integrated circuits as well as applications in artificial neural networks, pattern recognition, and image processing [25,26,27,28]. In recent developments, substantial progress has been made in the architecture of memristor-based neural network computing [29,30,31]. Wang et al. has developed a convolutional neural network with five levels that can adjust to the imperfections of the one-transistor–one-memristor array in order to accurately classify the MNIST dataset [32]. An experimental demonstration showing that a memristor crossbar array can be utilized to implement synaptic weights shared at different time steps in an LSTM [33]. This innovative approach not only minimizes circuit space requirements but also enables the storage of a vast number of parameters. Kong et al. introduced a novel 5D fractional order differentiation memristive Hopfield neural network (FOMHNN) [34]. Additionally, they presented a framework for constructing 2n + 1 dimensional simplest Hamiltonian conservative chaotic systems (HCCSs) to optimize encryption speed [35]. Furthermore, the capacity to do computations in memory is utilized, which successfully overcomes the constraints imposed by the ‘von Neumann bottleneck’. The neural signal analysis system’s high-efficiency capabilities allow for accurate neural signal identification and filtering associated with epilepsy. In addition, it shows a nearly 400% improvement in energy efficiency over CMOS technology. Different materials and structures have been utilized to develop a range of memristor devices following the example set by HP memristors [36,37,38], accompanied by the creation of numerous mathematical models for memristors. In particular, the VTEAM model (Voltage Threshold Adaptive Memristor) [39] is an appropriate option for memristor-based neuromorphic systems due to its fundamental physical device properties, experimental verification, and relatively straightforward and precise equations. Hence, our research is focused on utilizing VTEAM, which comprises two layers of TiO_2_-TiO_2_-x film positioned between independent Pt electrodes. It should be emphasized that there exist several other memristors and models demonstrating similar characteristics that can also be employed in this investigation. In particular, the utilization of a 1T1R configuration was proposed in this paper, which consists of a single transistor and memristor connected in series [40], revising the matter of leakage current that arises when applying a signal to an individual memristor. Furthermore, we incorporate image de-raining technology into the memristive neural network.

The main contributions of this study could be summarized as follows:

(1) The proposed memristive attention recurrent residual generative adversarial network (MARR-GAN) presents a software and hardware co-design of the image raindrop removal network, which leverages a memristor crossbar array and deep learning technology.
(2) A novel raindrop removal module is designed to construct a network for removing raindrops using the autoencoder architecture. Temporal changes in raindrops within images are effectively captured by this framework while preserving their location and shape information. Furthermore, an attention gate design is introduced to enhance the network’s understanding of both global structure and local details in order to achieve comprehensive raindrop removal across different regions of the image.
(3) A hardware implementation scheme utilizing memristor crossbar arrays for MARR-GAN is presented, integrating deep learning algorithms with neuron computing chips to enhance the speed of processing image data in the real world. Finally, the efficacy and superiority of MARR-GAN have been demonstrated in removing raindrops and restoring fine-grained image details.

## 2. Related Works

### 2.1. Rain Streaks Removal

Rain streaks removal can be classified into two distinct categories: solutions based on data analysis and solutions based on mathematical models. Zhuang et al. has presented a novel approach for rain removal by using a deep convolutional neural network [41]. Data-driven approaches involve techniques such as sparse coding, low-rank approximations [42], and Gaussian mixture models (GMMs). A rain-removal technique based on directional gradient priors has been proposed by Huang et al. [43]. Its goal is to eliminate the rain streaks while preserving as much of the original rain image’s structural information as feasible. On the other hand, a wavelet-based sparse optimization approach has been offered by Sun et al. [44] as a solution to the problem of the rain removal process, followed by joint optimization to remove rain-line details from the former while removing the rain line from the latter. Model-based methods encompass convolutional neural networks, transformers, and diffusion modules. Wan et al. has developed a global–local transformer, which employs window-based local transformer blocks to process high-resolution feature maps in shallow layers of GLFormer [20].

### 2.2. Snow Removal

Snow removal techniques commonly employ prior models based on snow driving features such as a histogram of oriented gradient (HOG) [45], color assumptions [46], and frequency spatial separation. Furthermore, the application of deep convolutional neural networks (CNNS) [47] has been increasingly employed in the domain of removing snow from individual images. These methods generally exhibit commendable performance owing to the resemblance in the appearance of snow particles.

### 2.3. Raindrop Removal

Currently, deep learning methods are predominantly employed for image raindrop removal tasks owing to their robust feature-extraction capabilities.

Qian et al. has integrated attention mechanisms into a type of generative adversarial network, known as AGAN, for capturing details related to both raindrops and their surroundings [14]. In order to improve raindrop-removal performance, a modular dual residual connection network named DURN for achieving high-quality image restoration outcomes has been proposed [48]. It is important to consider that variations in blur and resolution occurring due to differences in the distance between rainfall and the camera when observing rainy scenes within images. To tackle this issue, a network effectively handles diverse raindrop appearances by independently processing different frequency bands was proposed by Zini et al. [9]. It is capable of performing raindrop removal on images of varying scales, resulting in rapid and high-quality restoration. However, it necessitates substantial computational resources during training and exhibits prolonged processing time.

## 3. Model Design of MARR-GAN

### 3.1. Comprehensive Network Architecture

**Figure 1:**
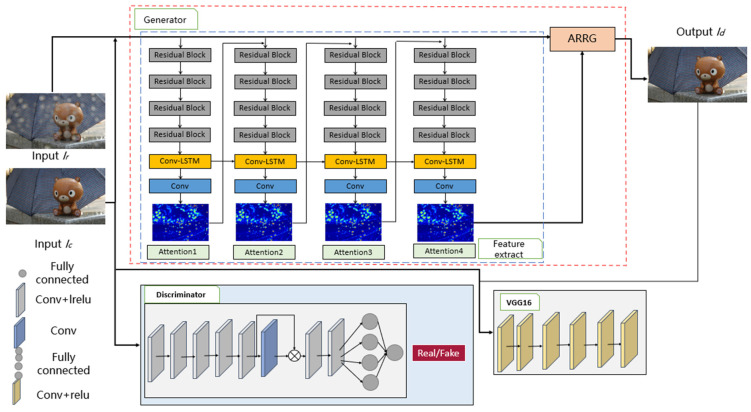
The network overview of MARR-GAN.

A one-stage network is proposed in this study for effectively separating the observed raindrop image *I* into clear the background *B* and raindrop *R*. The limitations of current progressive processing schemas on edge devices are being addressed, and the application and deployment of rain removal technology are being facilitated by this network. The mathematical expression could be represented by Equation (1). The network architecture of MARR-GAN is illustrated in Figure 1, where an autoencoder framework and attention gate connections are utilized as the raindrop removal network.

(1) I=(1−M(x))⊙B+R

where *M* is the mask, and *I* is defined as the input image. In this mask, *x* is a pixel of input image, *M*(*x*) is dependent on the variable *x*, and *M*(*x*) *=* 1 indicates that *x* represents the raindrop region, *M*(*x*) *=* 0 indicates the background of the input image. Furthermore, *B* stands for the background information and *R* represents mixed raindrops, which symbolize an intricate blend of previous knowledge and reflected light from the environment.

From Figure 1, a generative adversarial network (GAN) is considered to be the main body of this framework. The architecture consists of two sub-networks, namely a generator and discriminator. First, the blue box is the raindrop feature extraction module, which is used to extract raindrop features and generate attention maps. Furthermore, attention residual recurrent gate (ARRG) is guided to generate a raindrop-free image based on the generated attention map. Finally, the discriminator determines whether the input image originates from the generative network or the input image (i.e., *Ic*).

#### 3.1.1. Raindrop Feature Extraction Module

The visual attention model utilizes the attention recurrent network to enhance target recognition accuracy by identifying the target area [49]. Inspired by that, the network architecture employed in this paper bears resemblance to that designed for raindrop removal. The raindrop localization in an image is facilitated by the utilization of a visual attention guidance generative network and a distinguish network in our study. The generator section (see Figure 1) attention recurrent network comprises multiple circulation modules. There are four residual blocks, a convolution long short-term memory (Conv-LSTM) unit, and a convolution layer for each module. The residual block extracts raindrop feature information from both the input image and the previously generated attention map. Meanwhile, the Conv-LSTM unit and convolution layer generate a 2D attention map.

The generation of attention maps heavily relies on the mask, comprising only two distinct numerical values: 0 and 1. A value of 0 indicates the absence of raindrops in a pixel, while a value of 1 signifies the presence of raindrops in that pixel. The initial attention maps are generated by the first recurrent module of the attention cycle network, which takes both the raindrop image and the mask image as inputs. The mask map is obtained through the subtraction of the clear image from the raindrop-infused image, followed by the application of a specific threshold value for precise filtration. Despite its relatively coarse nature, the obtained mask map significantly contributes to generating a precise attention map. The main difference between the attention map and the mask map lies in what they contain: while the mask map solely consists of 0 and 1, the attention map values range from 0 to 1. The attention map’s higher median value indicates a stronger demand for pixel-focused attention, suggesting an increased probability of raindrops being present at that specific pixel. Even within identical areas affected by raindrops, variations could be observed in the attention map values due to differences in raindrop shape and thickness, thereby reflecting how raindrops impact different pixels within background images.

Conv-LSTM is a module that is part of the attention recurrent network. This module is composed of an input gate (named “*i_t_*”), a forgetting gate (called “*f_t_*”), an output gate (called “*O_t_*”), and a cell unit (called “*C_t_*”). The definition of the state-gates temporal interaction is given as follows:

(2) $$\left\{ \begin{array}{l} i_{t}=\sigma \left(W_{xi}*x_{t}+W_{hi}*H_{t-1}+b_{i}\right)\\ f_{t}=\sigma \left(W_{xf}*x_{t}+W_{hf}*H_{t-1}+b_{f}\right)\\ C_{t}=f_{t}•C_{t-1}+i_{t}•tanh\left(W_{xc}*x_{t}+W_{hc}*H_{t-1}+b_{c}\right)\\ O_{t}=\sigma \left(W_{xo}*x_{t}+W_{ho}*H_{t-1}+b_{o}\right)\\ H_{t}=O_{t}•tanh\left(C_{t}\right) \end{array}\right.$$

where the convolution operation is denoted by ∗, while element-by-element multiplication is represented as •. The Sigmoid function is defined as *σ*, and $x_{t}$ refers to input features. Weight matrix *W* corresponds to each convolution, and Tanh activation function is indicated by tanh. Bias vectors are represented as $b_{i}$, $b_{f}$, $b_{o}$. By storing threshold weights, Conv-LSTM effectively preserves raindrop pattern features and utilizes *H_t_* for hidden-layer representation. To perform computer vision tasks efficiently and adaptively, Conv-LSTM replaces fully connected weights in traditional LSTM with convolutional counterparts. Additionally, $H_{t-1}$ provides insights into determining the learning states of raindrops.

#### 3.1.2. Raindrop Removal Network

**Figure 2:**
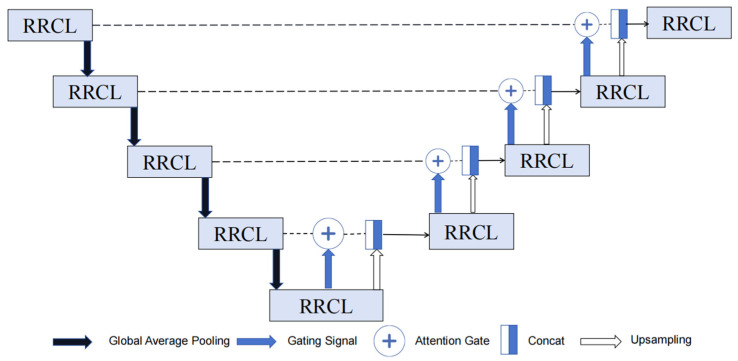
The structure of ARRG.

The raindrop removal network is an autoencoder, referred to as the attention residual recurrent gate (ARRG) module, illustrated in Figure 2.

Recurrent convolutional block combined with the residual unit is employed in both encoding and decoding paths. Additionally, attention gate (AG) is utilized instead of skipping connections to rectify low-resolution features by leveraging deep features. Moreover, batch normalization (BN) is incorporated to enhance the stability and accelerate the convergence speed throughout the neural network’s up-sampling procedure. The utilization of BN enables data standardization, facilitates smaller regularization, diminishes generalization errors, and enhances overall network performance [50].

**Figure 3:**
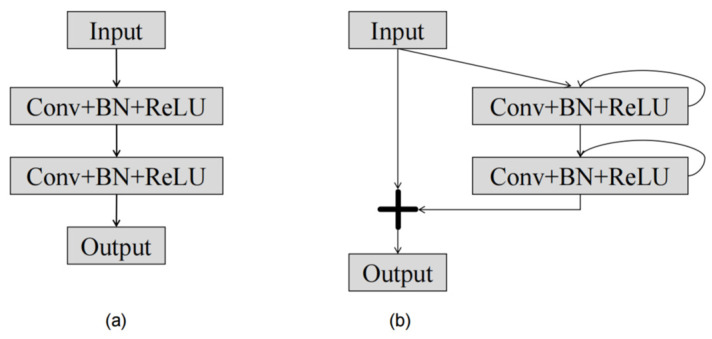
(**a**) The basic unit of the auto encoder convolution. (**b**) The structure of RRCL.

The autoencoder receives as input the attention map that the raindrop picture and the attention recurrent network produce. The raindrop removal and background restoration processes are guided by the attention map. The autoencoder consists of nine residual recurrent convolution layer (RRCL). Both decoding and encoding have a symmetrical structure. To avoid blurring the image, an attention gate connection is made between the corresponding modules. The detailed configuration of RRCL can be observed in Figure 3.

The RRCL block performs recurrent convolutional layers based on the discrete time steps described in the recurrent convolutional neural network [51]. Let us consider an input sample *u_l_* in the *l*th layer of the RRCL block and a pixel located at (*i*, *j*) within an input sample on the *k*th feature map in the recurrent convolutional layers. The output at time step *t*, denoted as $M_{ijk}^{l}\left(t\right)$, could be represented as follows:

(3) $$M_{ijk}^{l}\left(t\right)=\left(w_{k}^{f}\right)u_{l}^{f\left(i,j\right)}\left(t\right)+{\left(w_{k}^{r}\right)}^{T}u_{l}^{f\left(i,j\right)}\left(t-1\right)+b_{k}$$

In Equation (3), $u_{l}^{f\left(i,j\right)}$(*t*) and $u_{l}^{f\left(i,j\right)}$(*t* − 1) represent standard convolutional layers and the input sample of the *l*th recurrent convolutional layers, respectively. The standard convolutional layer and the recurrent convolutional layers of the *k*th feature maps are, respectively, weighted by $w_{k}^{f}$ and $w_{k}^{r}$, and *b_k_* is the bias. The output of recurrent convolutional layers is activated by standard ReLU function *f* as follows:

(4) $$F\left(u_{l},w_{l}\right)=f\left(M_{ijk}^{l}\left(t\right)\right)=\left(0,M_{ijk}^{l}\left(t\right)\right)$$

The output of the RRCL unit could be calculated as follows:

(5) $$u_{l+1}=u_{l}+F\left(u_{l},w_{l}\right)$$

where *u_l_* is an RRCL layer input sample. The output of the down-sampling layer in the encoding path and the output of the up-sampling layer in the decoding path are both represented by *u_l+1_*, respectively. Figure 3a depicts the fundamental unit of autoencoder convolution, while Figure 3b shows the RRCL block’s structure.

**Figure 4:**
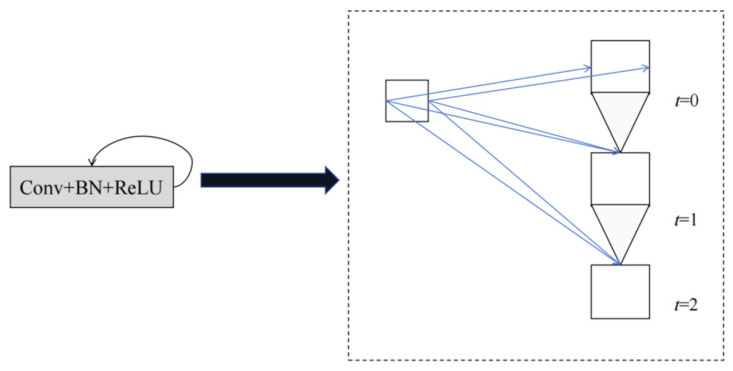
Unfolded recurrent convolutional units for *T =* 2.

Equations (4) and (5) depict the dynamic properties of recurrent convolutional layers. By extending the recurrent convolutional layers to *T* time steps, we can obtain a feedforward subnetwork with a depth of *T +* 1. In this study, we expand recurrent convolutional layers to two steps, denoted as *T =* 2. The structure of the expanded recurrent convolutional layers consists of a convolutional layer and two subsequent recurrent convolutional layers. Figure 4 shows the structure of the recurrent convolution layer.

### 3.2. Discrminator Network

The primary responsibility of the discriminative network is to discern between authentic and counterfeit data. In GAN, a global discriminator is typically employed by the discriminator to assess the disparity between the generated image and an authentic sample. However, relying solely on global information to evaluate the authenticity of an image prevents the producing network from effectively restoring local image details. In order to make following the target detection tasks easier, it is important to restore complex image elements as much as possible for raindrop removal. Consequently, existing discrimination networks cannot be directly utilized. Hence, both global and local discriminators are combined in this study to collectively ascertain whether output samples from the generator are true or false.

The local discriminator is employed by taking advantage of the information on the locations of the raindrops in the image. The attention map solves the problem of raindrop placements by being formed in the attention cycle network during the image restoration step. Consequently, the local discriminator could be guided to automatically recognize and pinpoint areas containing raindrops in a picture by adding the attention map into the discriminator network. Convolutional neural networks (CNNs) are used to extract features from the generator’s raindrop images as well as the discriminator’s inner layers. These extracted features are then combined with attention images to form a loss function for training the local discriminator. By introducing an attention map, it directs more focus towards discriminating raindrop regions within an image. In order to determine authenticity, a fully connected layer is utilized in conjunction with other layers in this discrimination network architecture as depicted in Figure 1’s lower section.

### 3.3. Loss Function

The generator and discriminator loss functions are contained in the MARR-GAN network. The generator loss function includes attention recurrent loss, raindrop-removal loss, and vgg perceptual loss. Both the global discriminator loss function and the attention map loss are included in the discriminator loss function.

Two images of the same backdrop scene, one with a raindrop and the other without, are fed into the generator. The loss function for each attention recurrent module is defined as the average squared difference between the output attention map and the binary mask, *M*. In the case of a recurrent network, a smaller weight is assigned to the loss function of the front module, while a larger weight is given to the back module’s loss function. The specific formulation of this loss function named *L_a_* could be observed in Equation (6).

(6) $$L_{a}\left(\{A\},M\right)=\sum_{t=1}^{N}\theta^{N-t}L_{MSE}\left(A_{t},M\right)$$

where *A_t_* represents an attention graph produced by a cyclic network at time step *t*. *A* represents the output attention map. *A_t_ = attention_t_*(*F_t−_*_1_, *H_t−_*_1_, *C_t−_*_1_), *F_t−_*_1_ denotes merging of raindrop-infused image and previous recurrent unit’s output attention map. Function *attention_t_* represents the attentive-recurrent network at time step *t*. With this recurrent network architecture, increasing value of N leads to the generation of more detailed attention maps; however, it also requires greater memory capacity for storing intermediate parameters. Optimal network efficiency has been observed when *N =* 4 and *θ =* 0.8.

There are two types of loss functions employed in the raindrop-removal loss, namely multiscale loss and perceptual loss. The multiscale loss function effectively utilizes image information from various decoder layers to enhance the model optimization process and generate raindrop free images with improved clarity. The multiscale loss function is defined as:

(7) $$L_{S}\left(\{I_{c}\},\{S\}\right)=\sum_{i=1}^{M}\lambda_{i}L_{MSE}\left(I_{ci},S_{i}\right)$$

where *S* represents the output of ARRG. *S_i_* represents the image characteristics that were extracted from the ARRG *i*-th layer, *I_c_* represents the input raindrop free image, and *I_ci_* indicates the ground truth that has the same scale as that of *S_i_*. ${\{\lambda_{i}\}}_{i=1}^{M}$ is the weight of different scales. Large-scale picture feature extraction is given more consideration in the design of the loss function, whereas smaller images have less information and hence minimal bearing on model optimization. The last layer, third layer, and fifth layer of the decoder have output picture sizes of 1/4, 1/2, and 1 of the original sizes, respectively. The weights *λ* associated with these sizes are set to 0.6, 0.8, and 1.0.

In addition to the loss based on pixels, perceptual loss is also incorporated in this study, to assess the overall difference between the output of an automatic context encoder-decoder and its corresponding clear image. Perceptual loss evaluates how similar a raindrop-free image is to its real counterpart from a global perspective, thereby bringing it closer to reality. The *VGG*_16_ model could extract global information from images but requires pretraining on an ImageNet dataset beforehand. The definition of perceptual loss function is as follows:

(8) $$L_{p}\left(I_{d},I_{c}\right)=L_{MSE}\left(VGG_{16}\left(I_{d}\right),VGG_{16}\left(I_{c}\right)\right)$$

where the *VGG*_16_ model is a pre-trained convolutional neural network (CNN) that performs feature extraction on an input image. $I_{d}$ represents the output image generated by the automatic encoder, denoted as $I_{d}=G\left(I_{r}\right)$, while $I_{r}$ refers to a real-image sample devoid of raindrops. In summary, the generator network’s loss function could be explained as follows:

(9) $$L_{G}=10^{-2}L_{GAN}\left(I_{d}\right)+L_{a}\left(\{A\},M\right)+L_{S}\left(\{S\},\{A\}\right)+L_{P}\left(I_{d},I_{c}\right)$$

where $L_{GAN}\left(I_{d}\right)=\log\left(1-D\left(I_{d}\right)\right)$.

The whole loss function of the discrimination network could be expressed as

(10) $$L_{D}\left(I_{d},I_{r},A_{t}\right)=-\log\left(D\left(I_{r}\right)\right)-\log\left(D\left(I_{D}\right)\right)+\alpha L_{map}\left(I_{d},I_{r},A_{t}\right)$$

where α is 0.05, the global discriminator’s loss function is represented by the first two components of the formula, the local discriminator’s loss function is represented by *L_map_*, and the local discriminator’s loss function is displayed as follows:

(11) $$L_{map}\left(I_{d},I_{r},A_{t}\right)=L_{MSE}\left(D_{map}\left(I_{d}\right),A_{t}\right)+L_{MSE}\left(D_{map}\left(I_{r}\right),0\right)$$

where *D_map_* is a 2D attention mask function generated by the discriminator, while $I_{r}$, $I_{d}$ represents a pair of image samples extracted from the database. A value of 0 in the attention map indicates the absence of raindrops in the real image, hence no guidance is needed for feature extraction. The discriminator architecture in this study comprises eight convolution layers with a core size of (3, 3), followed by a fully connected layer with 1024 neurons that employ Sigmoid activation.

## 4. Memristive Circuit Design of MARR-GAN

In this section, a memristor circuit implementation scheme for MARR-GAN is proposed. To reduce training time costs, we pre-determine the network parameters using existing training and then map them onto the crossbar array. We utilize linear mapping to convert input data into an analog voltage signal that is scaled by an operational amplifier with a factor of 1000 to adjust the voltage from *V* to *mV*. This ensures that all feature information of the input circuit meets the memristor’s voltage threshold. Based on the VTEAM memristor’s threshold characteristic, when reading signals that are lower than writing-threshold voltage, there is minimal change in memristor resistance, thus, ensuring stable storage of network parameters in the crossbar array during the de-raining process. Finally, pixel-value information is recovered by reconstructing output results from the memristive neural network. In this section, we will provide further details regarding implementing three foundational components of MARR-GAN using a memristive circuit approach.

### 4.1. Convlution Calculation Implementation

**Figure 5:**
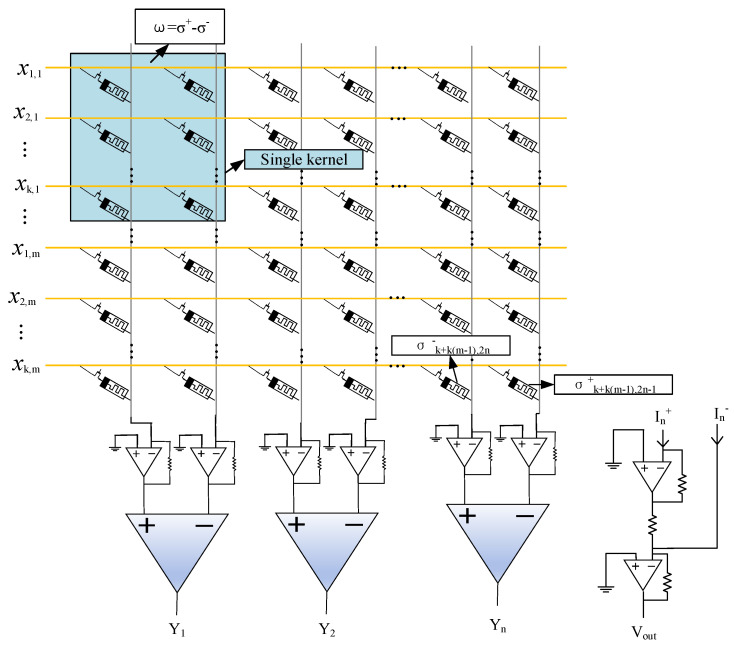
Template for implementing a memristive circuit to perform convolution calculations.

In order to preserve the input edge information and avoid a decrease in output size, it is common practice in software programs to employ the same padding technique. This involves padding the input feature maps with zeros before performing convolution operations, ensuring that the output size remains unchanged. In terms of implementing crossbar arrays, this can be achieved by adding zero values to the input signals. As convolution kernel weights can encompass positive, negative, and zero values, whereas meminductance is restricted to non-negative values only, it is not feasible to directly represent negative values using a single memristor. Hence, two memristors are needed to store a single weight value. Assuming *m* and *n* represent the count of input and output channels, respectively, while k represents the dimension of convolution kernels. The corresponding memristive circuit configuration for this purpose is illustrated in Figure 5. The matrices of mk*1 and mk*2 are used to represent the inputs and convolution kernel matrices, respectively. The input voltage signals are transmitted horizontally and multiplied by the conductance. By leveraging Ohm’s law for multiplication and Kirchhoff’s current law for accumulation, the total currents are collected vertically. The two collected vertical currents are first passed through an amplifier to increase the amplitude of the signal, making the summed output more obvious, and then the two amplified currents are collected through the summing amplifier (blue block). The circuit in Figure 5 could be described using Equation (12). Each input-feature map undergoes *n* convolution operations, followed by m*n convolution operations on *m* maps. Consequently, *n* groups of *m* output maps need to be merged together to obtain the final *n* result maps. In other words, two columns perform single feature extraction.

(12) $$Y_{N}=R_{L}\left[\sum_{m=1}\sum_{k=1}x_{k,m}•\left(\sigma_{k+k\left(m-1\right),2n-1}^{+}-\sigma_{k+k\left(m-1\right),2n}^{-}\right)\right]\left(n=1,2,3\ldots \right)$$

The input voltage signal is denoted as *x_k_*_,*m*_, while $\sigma^{+}$ and $\sigma^{-}$ correspond to the conductance of a pair of memristors. It should be noted that all instances of $\sigma^{-}$ have predetermined fixed values for their conductance. Therefore, by adjusting the value of $\sigma^{+}$ through calibration, we can effectively represent the convolution kernel weights associated with positive, negative, and zero.

The input to the network in this paper is an RGB, three-channel image with dimensions of *W × H* and a convolution kernel size of 3 × 3. Consequently, the image is transformed into an output vector of size *W × H ×* 1, and each time a 3 × 3 field is added. This vector is then sent to the convolution kernel for the convolution operation. During this process, the convolution kernel continuously slides over the input image with a fixed step size. The convolution operation involves calculating the sum of products between shared weights associated with the convolution kernel and the generated input image. This operation could be decomposed into parallel multiply-accumulate operations, making it suitable for memristor-based in-memory computing architectures. To facilitate hardware implementation, the input image is expanded into a nine-dimensional vector, which drives a corresponding nine pulse channels to provide simultaneous access to nine-bit lines. The weights are represented by two differential conductances of 1T1R memristors, mapping positive and negative rows, respectively, for each weight value within a given convolutional kernel. By doing so, we calculate differences between cumulative flowing currents on two relevant source lines as weighted sums of both convoluted inputs and their respective kernel weights.

### 4.2. RRCL Network

MARR-GAN consists of the RRCL module and the global average pooling layer attention gate. Each RRCL module is followed by a BN processing by each convolution kernel, and the nonlinearity is introduced by ReLU activation function, after which it is passed to the next convolution layer.

#### 4.2.1. Global Average Pooling Module

The global average pooling layer module (GAP) pools feature maps of different input sizes into 1 × 1 feature maps. The module will perform a global average pooling operation on images of size C*H*W. Therefore, instead of using memristors with varying resistance values, a resistor is used instead. Memristors are used in the summation circuit, and average pooling is implemented by a column parallel memristor cross array. All feature information within the pool window (one pool) is fed to the parallel cross array, which could be expressed as:

(13) $$VA_{t}=\sum_{r}\left(\frac{V_{C,HW}\times R}{M_{t}}\right),t=1,2,3\ldots n$$

**Figure 6:**
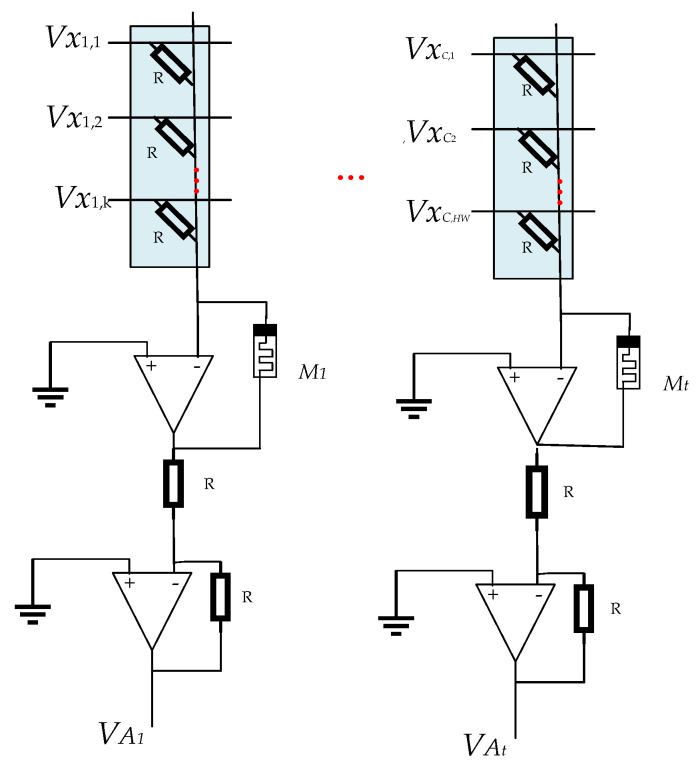
Realization of the memristive circuit for calculating GAP.

The schematic diagram of the GAP is illustrated in Figure 6. Assuming the pooling window size is *k*, The memristor’s significance within the module for an average pooling circuit $M_{t}=k\times R$, where *R* represents resistance. It should be noted that *R* is set to 1 kΩ.

Since the GAP layer does not require training weights in its computation, it takes a simplified approach to processing data. Specifically, it utilizes resistors instead of memristors that require training weights, which are determined during the manufacturing process and do not need to be trained or adjusted at runtime. This design greatly simplifies the complexity of the circuit and improves computational efficiency.

However, the summation circuit part is different. Here, the nonlinear nature of the memristor plays a key role. Due to this property of the amnesia, it can efficiently handle massively parallel computing tasks, which is crucial when dealing with large amounts of data. By applying the amnesia to the summation circuit, we can achieve fast and accurate data processing, which is crucial for the operation of neural networks.

In summary, the design of the GAP layer using a cross array of amnesia on the circuit as well as a simple summation circuit is an innovative approach. It skillfully combines the memory properties of the memristor and the stability of the resistor, making the whole circuit both simple and efficient.

#### 4.2.2. Batch Normalization (BN)

The operation of the BN layer can be simplified as a linear transformation of the input *x.* The calculation method is as follows:

(14) y=ax+b

A circuit for achieving a linear variation in input *x* can be realized by integrating an operational amplifier with two memristors.

**Figure 7:**
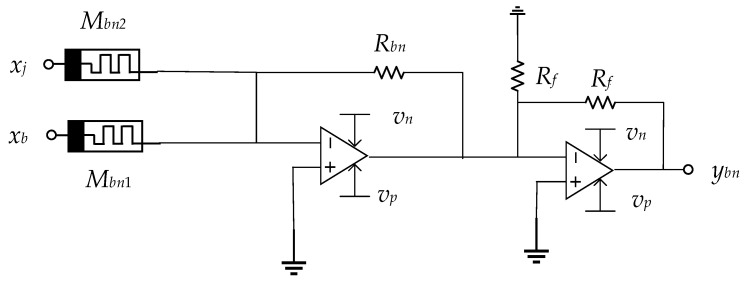
Realization of the memristive circuit for calculating BN.

The schematic diagram of the BN layer is illustrated in Figure 7. The op amp on the right functions as an inverting adder circuit, generating the output signal as follows:

(15) $$y_{bn}=\frac{R_{bn}}{M_{bn1}}x_{j}-\frac{R_{bn}}{M_{bn2}}x_{b}$$

The primary function of the BN is to adjust the distribution of input feature data *x*. Therefore, it is essential for the transformation coefficient a of the BN layer to be positive. *R_bn_*, *M_bn_*_1_, and *M_bn_*_2_ are greater than 0, if and only if *x_b_ >* 0, *b <* 0.

Of the inputs *x_j_* and *x_b_*, *x_j_* is the input data, *x_b_* is the bias input, where *x_j_* and *x_b_* are input into two memristors, and then input into the inverting operational amplifier circuit to perform linear transformation to obtain the output *y_bn_*. The principle of this design is to use the memristors to store xi, and then through the inverting operational amplifier circuit to perform the linear transformation to realize the linear amplification or reduction in the input signal. The principle of this design is to use a memristor to store *x_j_*, and then perform a linear transformation through an inverting operational amplifier circuit to linearly amplify or reduce the input signal. By changing the conductance value of the memristor and adjusting the behavior of the BN layer, this design can improve the training efficiency and stability of the neural network and reduce the sensitivity of the model to the initial weights.

#### 4.2.3. Sigmoid

Based on the exponential circuit, we have successfully developed circuits for implementing the activation functions sigmoid. The resulting outputs could be mathematically represented as follows:

(16) $$V_{s}=\frac{V_{b}}{V_{b}+\exp\left(V_{i}\right)}$$

where *V_b_* represents a bias voltage with a fixed size of 1 V.

**Figure 8:**
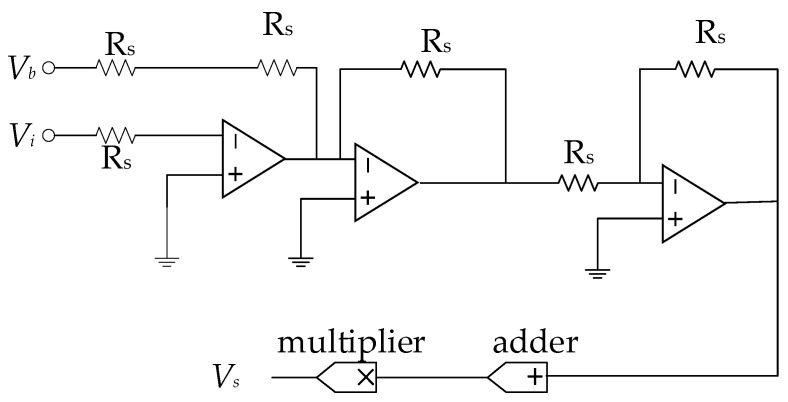
Realization of the memristive circuit for calculating Sigmoid.

As the circuit shown in Figure 8, *V_b_* represents the bias voltage, *V_i_* represents the input voltage, and *V_s_* represents the output voltage, which is realized by three inverting amplifier circuits, an adder, and a multiplier. A bias voltage of 1 V is added to the input of this circuit, as the sigmoid function changes its output slowly when the input is close to zero. By adding a bias voltage of 1 V, the input voltage can be made to vary over a wider range, which improves the linearization of the sigmoid function and extends the linearization range of the sigmoid function. In the neural network designed in this paper, this helps to improve the linear response of the network to the inputs, allowing the network to better learn and recognize the features of various raindrops.

## 5. Experiments

The performance and superiority of MARR-GAN are validated on various artificial datasets and real-world images affected by raindrops, with qualitative and quantitative comparisons conducted against different state-of-the-art (SOTA) techniques.

### 5.1. Implementation Details

#### 5.1.1. Datasets

There are two types of datasets utilized in the experiment. One dataset was obtained by Qian et al. [14] through the manual application of water droplets on the camera lens followed by capturing photos. A total of 861 pairs of training data constitutes this dataset, comprising images with raindrop effects and their corresponding clean versions. The test dataset comprises Testa and Testb, containing a total of 58 and 249 samples, respectively. The other dataset that Quan et al. [52] constructed is a comprehensive dataset of real raindrop images, consisting of 1000 high-resolution photographs captured with a single-lens reflex (SLR) camera. A total of 250 pairs of raindrop images are encompassed by this dataset, covering diverse environmental settings such as parking lots, parks, and urban areas. Moreover, data collection were conducted at different times of the day morning, noon, and afternoon to capture raindrop images under varying lighting conditions in authentic scenes.

#### 5.1.2. Training Details

The proposed network is trained and tested on Ubuntu 20.04, 128 GB host memory, 32 GB GPU, CUDA 11.6, CUDNN 8.5, Python 3.9, and NVIDIA RTX-3090. The experimental condition is based on Pytorch-GPU 1.12.0. The size of the input image pair is 720 × 480. We used the Adam optimizer with a learning rate of 0.0001 and a decay rate of 0.90. The model was trained for 500 epochs, and the entire training time of the proposed model was approximately 30 h.

### 5.2. Experimental Results

The Raindrop and RainDS datasets are utilized in this experiment to evaluate the performance of the network, which is then compared with state-of-the-art raindrop removal networks including AGAN [14], ATT [53], A2Net [54], DURN [48], and TMN [55]. By comparing the above networks, the raindrop removal performance is evaluated from SSIM, PSNR indicators, and visual effects.

#### 5.2.1. Quantitative Evaluation

The evaluations on the Raindrop dataset of our approach are presented in Table 1 in comparison with AGAN [14], ATT [53], A2Net [54], DURN [48], and TMN [55].

From the comparison in Table 1, it can be seen that in contrast to the previous best approach, MARR-GAN improves 0.005 of SSIM on Testb; MARR-GAN improves 0.148 dB of PSNR on Testa and improves 0.397 dB of PSNR on Testb; MARR-GAN improves 1 of PHah on Testa.

The evaluations on the RainDS dataset of our approach are presented in Table 2 in comparison with AGAN [14], ATT [53], A2Net [54], DURN [48], and TMN [55].

From the comparison in Table 2, it can be seen that in contrast to the previous best approach, MARR-GAN improves 0.023 dB of PSNR and 0.023 of SSIM.

#### 5.2.2. Qualitative Evaluation

**Figure 9:**
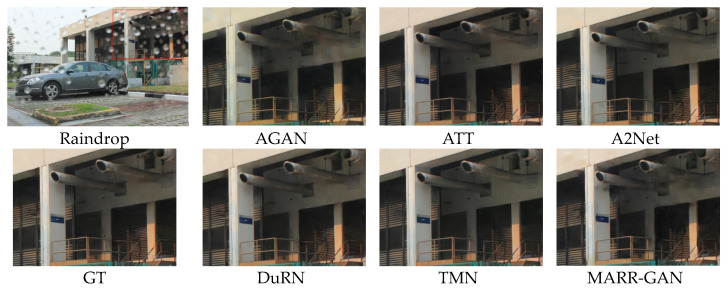
Qualitative evaluation on Testa.

**Figure 10:**
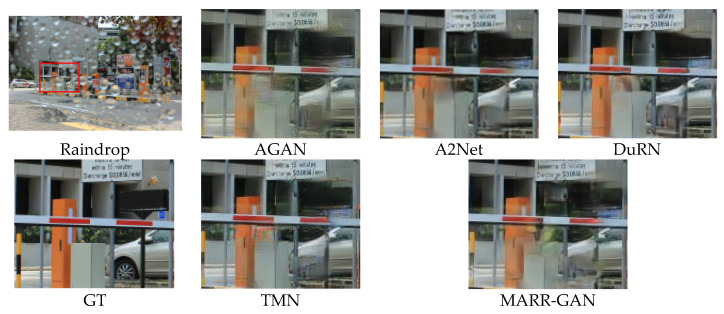
Qualitative evaluation on Testb.

**Figure 11:**
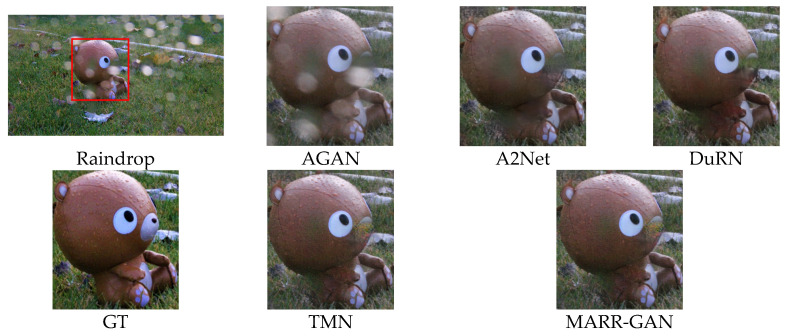
Qualitative evaluation on RainDS.

The visual comparison results of various raindrop removal methods are depicted in Figure 9 and Figure 10. It can be observed that AGAN [14] has a color shift. The methods ATT [53] and DuRN [48] are associated with the presence of conspicuous distortions in terms of both artifacts and color accuracy. Although A2Net [54] and TMN [55] demonstrate satisfactory performance in overall perception, they lack consistency when it comes to restoring local structures from pristine samples. On the contrary, our approach guarantees the retention of authentic background details without any visually perceptible distortions. Moreover, Figure 11 presents a comparative analysis showcasing raindrops exhibiting reflected colors at different transparency levels. It becomes apparent that existing techniques yield restored images containing undesirable artifacts or incomplete semantic information. In contrast, our method enables more dependable image reconstruction while effectively accommodating various raindrop morphologies.

### 5.3. Ablation Study

This section develops an ablation study to investigate the importance of various MARR-GAN components. All of the approaches’ ablation experiments are carried out on Raindrop. In particular, we created the four variation experiments listed below:

Experiment I: In this experiment, we will exclude the recurrent convolutional unit and add a connection line in RRCL, instead utilizing the conventional convolution layer depicted in Figure 3b for image rain removal processing.

Experiment II: For this experiment, we will replace the attention gate connections in RRCL with skip connection blocks.

Experiment III: For this experiment, we will change the number of residual blocks to three in the recurrent attention network.

In experiments I to III, we examined the impact of incorporating recurrent convolutional units and adding connection lines, attention gate connections in RRCL, and varying the number of residual blocks in the recurrent attention network on image de-raining outcomes. To ensure reproducibility and minimize training randomness, we calculated average result values using Raindrop datasets. Table 3 presents a quantitative comparison of these three variations. It is evident that our MARR-GAN consistently achieves higher PSNR and SSIM scores compared to the other variants. Conversely, our overall model effectively enhances structural details in the restored images.

## 6. Conclusions

A novel image de-raining network, named MARR-GAN, which combines software and hardware design, is presented in this study. The proposed network utilizes an attention recurrent residual generative adversarial network to effectively remove raindrops from images. Additionally, we introduce a cyclic raindrop feature extraction module that facilitates the analysis and interpretation of pixel relationships within the raindrop feature image. Then, a raindrop removal module is designed to handle image areas with complex texture details. Based on this premise, a memristor-based hardware implementation scheme is put forward, offering an advantageous hardware resolution for accelerating DCNN and ensuring real-time and dependable processing of intricate images. Experimental results show that the MARR-GAN algorithm proposed in this article has good application prospects in removing raindrops and restoring image details.

## Figures and Tables

**Table 1:** Results of Raindrop dataset provided by Qian et al. [14].

Dataset	Metric	AGAN	ATT	A2Net	DURN	TMN	MARR-GAN
Testa	PSNR (dB)	29.569	30.808	27.961	30.1008	30.062	30.956
	SSIM	0.905	0.917	0.911	0.912	0.905	0.900
	PHah	3	1	4	2	2	1
Testb	PSNR (dB)	24.228	-- ^1^	25.081	24.322	24.313	25.478
	SSIM	0.786	-- ^1^	0.793	0.802	0.787	0.807
	PHah	4	-- ^1^	2	3	4	2

^1^ “--”: results are not released.

**Table 2:** Results of RainDS dataset provided by Quan et al. [52].

Dataset	Metric	AGAN	ATT	A2Net	DURN	TMN	MARR-GAN
RainDS	PSNR (dB)	24.298	-- *	24.461	23.882	24.151	24.484
	SSIM	0.819	-- *	0.842	0.818	0.828	0.847
	PHah	4	-- *	3	4	4	3

* “--”: results are not released.

**Table 3:** Ablation results of Raindrop dataset provided by Qian et al. [14].

Dataset	Metric	Experiment I	Experiment II	Experiment III	MARR-GAN
Testa	PSNR (dB)	29.358	29.941	28.692	30.956
	SSIM	0.896	0.884	0.865	0.900
	PHah	3	3	4	1
Testb	PSNR (dB)	24.376	25.310	25.327	25.478
	SSIM	0.792	0.801	0.783	0.807
	PHah	2	2	2	2

## Data Availability

The data that support the findings of this study are available from the corresponding author upon reasonable request.
